# Advances in understanding itching and scratching: a new era of targeted treatments

**DOI:** 10.12688/f1000research.8659.1

**Published:** 2016-08-22

**Authors:** Kristen M. Sanders, Leigh A. Nattkemper, Gil Yosipovitch

**Affiliations:** 1 Department of Dermatology, Lewis Katz School of Medicine, Temple University, Philadelphia, PA, 19140, USA; 2 Department of Dermatology and Itch Center, Miller School of Medicine, University of Miami, Miami, FL, USA

**Keywords:** Pruritus, Mas-related G protein-coupled receptor A3, Gastrin-releasing peptide, astrogliosis

## Abstract

Chronic itch is a significant health burden with few effective treatments. As such, itch researchers seek to understand the mechanisms behind itch and to find potential targets for treatment. The field of itch research is dynamic, and many advances have been made so far this decade. In particular, major steps forward include the identification of new peripheral and central itch mediators and modulators, the discovery of greater roles for immune cells and glia in itch transmission, and a focus on the brain processing of itching and scratching. Finally, several new therapeutic interventions for itch have shown success in clinical trials.

## Introduction

Itch, or pruritus, affects between 8 and 38% of the general population worldwide and is a common feature of diseases such as atopic eczema, psoriasis, and uremia ^1^. Despite the prevalence of itch and its impact on quality of life, there are still many aspects of itch signal transmission that are unexplained, and treatments for chronic itch are lacking. However, the field of pruritus research is constantly changing and growing, and many areas of discovery have taken root in the six years since our previous F1000 report ^2^. There have been many exciting new finds in itch research in both humans and animal models that have both given us insight into the mechanisms of itch signaling and informed advances in treatment.

## Peripheral mediators of itch

Many recent studies have worked to define the roles of various itch mediators and receptors in the skin. Of these, the identification of an itch-specific population of neurons represents a major advance in the itch field. Mas-related G protein-coupled receptor A3 (MrgprA3) was previously identified to be a receptor for non-histaminergic itch, as it is activated by chloroquine (and not histamine) administration ^3^. Building on this research, Han *et al.* ^4^ identified an itch-specific population of MrgprA3-expressing neurons in the dorsal root ganglion (DRG). These neurons exclusively innervate the epidermis, respond to multiple pruritogens, and form synapses with gastrin-releasing peptide receptor-positive (GRPR ^+^) neurons in the dorsal horn of the spinal cord. Crucially, ablation of these neurons reduces scratching in response to pruritogen injection without altering pain responses.

Research into other members of the Mrgpr family in animal models has also been fruitful. The pruritogenic peptide SLIGRL (Ser-Leu-Ile-Gly-Arg-Leu) activates MrgprC11 ^5^, while β-alanine induces itch through MrgprD ^6^. Both MrgprA3 ^+^ and MrgprD ^+^ neurons were found to be hyperexcitable in a mouse model of contact dermatitis, suggesting a role for these neurons in chronic itch as well as acute itch ^7^. More importantly, some of these results have been validated in human ^8^ and monkey ^9^ studies.

Several members of the thermosensitive transient receptor potential (TRP) channel superfamily are expressed in normal human skin and have been implicated in itch. TRP vanilloid 1 (TRPV1) is necessary for histaminergic itch transmission ^10^, and TRP ankyrin 1 (TRPA1) was found to be a downstream target of MrgprA3 and MrgprC11 necessary for itch signaling through these receptors ^11^. Deletion of TRPA1 also abolished scratching in a dry skin mouse model of chronic itch, further cementing its role in non-histaminergic itch transmission ^12^.

Another peripheral mediator, endothelin-1 (ET-1), was found to induce both itch and pain behaviors in mice, as distinguished by the cheek injection model ^13^. ET-1 iontophoresis also induced itch in human subjects, and ET-1 and its downstream mediators were upregulated in patients with prurigo nodularis and chronic itch ^14^.

Voltage-gated sodium channel (NaV) 1.7, previously known to be involved in pain transmission, has also been found to mediate itch. A gain-of-function mutation in *SCN9A* (the gene encoding NaV 1.7) in three family members was associated with intense paroxysmal itch triggered mainly by warmth and spicy food ^15^. Furthermore, a monoclonal antibody targeting NaV 1.7 suppressed inflammatory and neuropathic pain as well as acute and chronic itch in mice, suggesting that this channel may be a target for both pain and itch treatment ^16^.

## Peripheral crosstalk between neurons and immune cells

Immune cells interact directly with nerve fibers in the skin, and this crosstalk has been shown to have important functions in pathological itch. Several pruritic diseases have been associated with increased levels of Th2 cytokines (for review, see 17), and interleukin-31 (IL-31) in particular has been identified as an “itchy” cytokine in atopic eczema. Recently, IL-31 was found to be produced by malignant T cells in cutaneous T cell lymphoma (CTCL), and serum levels of IL-31 correlated to CTCL pruritus severity ^18^. We examined expression levels of IL-31 and its receptors in the skin and found a correlation between epidermal expression and itch severity ^19^. Epidermal expression of IL-31 receptors was also found to be increased in the skin of patients with lichen amyloidosis and pruritus ^20^. In mice, both single and repeated intradermal administration of IL-31 caused an increase of scratching behavior, and repeated exposure led to increased expression of IL-31RA in the DRG ^21, 22^.

## Central itch transmission

Gastrin-releasing peptide (GRP) and its receptor (GRPR) were previously identified as the first itch-specific mediators in the spinal cord ^23^, and recent research has expanded on this pathway. Mishra and Hoon ^24^ reported that mice lacking natriuretic polypeptide b (Nppb), normally expressed in the DRG, showed almost no scratching in response to a range of intradermally injected pruritogens. These mice retained normal responses to touch, pain, and other stimuli, suggesting that Nppb ^+^ neurons are specific to itch transmission. Intrathecal injection of GRP still induced scratching in Nppb knockout animals, leading the authors to propose a model of itch signaling with Nppb and its receptor NPRA upstream of GRP-GRPR.

However, this study sparked controversy in the itch field. Following another school of thought, Liu *et al.* ^25^ rebutted that Nppb signaling does not occur upstream of the GRP-GRPR pathway. They were unable to replicate immunostaining of Nppb and NPRA and, in contrast to Mishra and Hoon, were able to detect *Grp* mRNA in the DRG. Additionally, they reported that blocking GRP-GRPR signaling did not alter Nppb-induced scratching. They argued that the role of Nppb remains to be clarified.

In addition to research into spinal transmission of the itch signal, there has been significant work on the inhibition of itch at the spinal level. Mice lacking Bhlhb5 ^+^ inhibitory (B5-I) interneurons in the spinal dorsal horn displayed increased scratching and subsequent skin lesions ^26^. Further experiments revealed that this population of B5-I interneurons is activated downstream of certain TRP channels and releases dynorphin, a kappa opioid receptor ligand, to inhibit itch ^27^. Additionally, a population of spinal interneurons expressing neuropeptide Y has been found to gate itch induced by light mechanical stimuli ^28^.

## Central interactions between neurons and glia

Previous studies demonstrated the involvement of glial cells in neuropathic pain, and recent work suggests their involvement in modulating itch. Signal transducer and activator of transcription 3-induced astrogliosis in the spinal dorsal horn was observed in mouse models of both contact dermatitis and atopic dermatitis ^29^. This activation was reduced in mice with clipped toenails but was still present in artificially scratched mice, suggesting that scratching plays a crucial role in this kind of reactive astrogliosis. Another study found that Toll-like receptor 4, part of the innate immune system, induced astrogliosis in the spinal dorsal horn of mice with dry skin itch ^30^. This astrogliosis was also dependent on scratching; preventing scratching with an Elizabethan collar also prevented astrogliosis. Of note, microglial activation was mild and transient in this model.

In contrast, an increase in microglial activation within the spinal cord was seen after intradermal injection of either compound 48/80 (a histaminergic pruritogen) or 5'-guanidinonaltrindole (a kappa opioid receptor antagonist) in mice ^31^. In a chronic itch mouse model, spinal microglia were activated after scratching, and suppression of microglia via minocycline treatment in turn reduced the level of scratching ^32^. A similar pattern was observed in a mouse model of atopic dermatitis. These mice displayed increased levels of microglia in the spinal cord, and minocycline treatment reduced scratching and improved dermatitis scores ^33^.

Chronic itch features not only spontaneous itch but also the dysethesias of alloknesis (itch evoked by light, normally non-itchy touch) and hyperknesis (increased itch in response to a normally itchy stimulus). These sensations are thought to be caused by central sensitization. Although these properties have been long reported in patients with chronic itch and in healthy skin injected with histamine, there was a lack of animal models to study them. Such models are especially important because the mechanisms behind this increased sensitivity appear to be related to, but distinct from, those underlying spontaneous itch. To this end, an elegant mouse model of alloknesis was developed by using von Frey filaments to stimulate mice with a light touch sensation ^34^. Mice displayed scratching in response to light touch after intradermal injection with several different pruritogens, as well as in a dry skin itch model.

## Stress and reward in the itch-scratch cycle

Itch is intrinsically linked with scratching, and the pleasure and relief evoked by scratching have been the focus of several recent studies. The intensity of itch (experimentally induced with cowhage) and the pleasure of scratching were found to vary according to the area of the body being scratched, and there was greater itch, itch relief, and pleasure during scratching of the back and ankle in comparison with the forearm ^35^. Functional magnetic resonance imaging using arterial spin labeling demonstrated that scratching an itch led to activation in several brain areas involved in reward and perception, including the striatum, midbrain, primary somatosensory cortex, and insula ^36^. We further found a difference in brain activity during active scratching of an itch by the participant compared with passive scratching by an experimenter ^37^. Several reward-associated areas (ventral tegmental area, nucleus accumbens, caudate nucleus, and ventromedial prefrontal cortex) were activated during active scratching and correlated with pleasure, itch relief, or both.

Greater attention is also being paid to the role of stress in itch and possible psychological and educational interventions. These techniques include habit reversal training, relaxation therapy, and cognitive behavioral therapy ^38^. Such interventions represent a unique approach to breaking the itch-scratch cycle and managing chronic itch. They can be helpful both for chronic itch patients and for caretakers of children with chronic itch.

## Novel targets for chronic itch

A revolution in the treatment of chronic pruritus is under way. Recently, much effort in treating has been placed on targeting the immune system in pruritic diseases. Agents that target IL-4 and IL-13 ^39^ and IL-31 ^40^, as well as a topical phosphodiesterase 4 inhibitor ^41^, have been used in patients with atopic eczema, whereas biologics that target IL-17 ^42^ and oral phosphodiesterase 4 inhibitors ^43^ have shown anti-pruritic efficacy in patients with psoriasis.

Agents targeting the nervous system are also showing promise in clinical trials for itch of different types. Nalfurafine, a kappa opioid agonist, has continued to be a successful anti-itch therapy ^44^, and other kappa opioid agonists and combined kappa opioid agonists/mu opioid antagonists are being explored as treatment options ^45^. Neurokinin 1 inhibitors have also shown some success in the treatment of itch ^46^.

Due to the complexity of the different etiologies, cell types, and mediators involved in chronic itch, a combination of topical and systemic therapies addressing peripheral mediators and top-down approaches targeting the brain and spinal cord may be the best strategy of treatment. The mediators identified in current animal and human studies may prove to be effective targets in future therapies.

## References

[1] Mollanazar NK Koch SD Yosipovitch G: Epidemiology of Chronic Pruritus: Where Have We Been and Where Are We Going? *Curr Derm Rep.* 2015;4(1):20–9. 10.1007/s13671-014-0093-y

[2] Yosipovitch G: Recent advances in pruritus - what we have learned and where are we headed. *F1000 Med Rep.* 2010;2: pii: 39. 10.3410/M2-39 20948846 PMC2950052

[3] Liu Q Tang Z Surdenikova L: Sensory neuron-specific GPCR Mrgprs are itch receptors mediating chloroquine-induced pruritus. *Cell.* 2009;139(7):1353–65. 10.1016/j.cell.2009.11.034 20004959 PMC2989405

[4] Han L Ma C Liu Q: A subpopulation of nociceptors specifically linked to itch. *Nat Neurosci.* 2013;16(2):174–82. 10.1038/nn.3289 23263443 PMC3557753

[5] Liu Q Weng HJ Patel KN: The distinct roles of two GPCRs, MrgprC11 and PAR2, in itch and hyperalgesia. *Sci Signal.* 2011;4(181):ra45. 10.1126/scisignal.2001925 21775281 PMC3144551

[6] Liu Q Sikand P Ma C: Mechanisms of itch evoked by β-alanine. *J Neurosci.* 2012;32(42):14532–7. 10.1523/JNEUROSCI.3509-12.2012 23077038 PMC3491570

[7] Qu L Fan N Ma C: Enhanced excitability of MRGPRA3- and MRGPRD-positive nociceptors in a model of inflammatory itch and pain. *Brain.* 2014;137(Pt 4):1039–50. 10.1093/brain/awu007 24549959 PMC3959553

[8] Sikand P Dong X LaMotte RH: BAM8-22 peptide produces itch and nociceptive sensations in humans independent of histamine release. *J Neurosci.* 2011;31(20):7563–7. 10.1523/JNEUROSCI.1192-11.2011 21593341 PMC3111068

[9] Wooten M Weng HJ Hartke TV: Three functionally distinct classes of C-fibre nociceptors in primates. *Nat Commun.* 2014;5: 4122. 10.1038/ncomms5122 24947823 PMC4072246

[10] Shim WS Tak MH Lee MH: TRPV1 mediates histamine-induced itching via the activation of phospholipase A _2_ and 12-lipoxygenase. *J Neurosci.* 2007;27(9):2331–7. 10.1523/JNEUROSCI.4643-06.2007 17329430 PMC6673467

[11] Wilson SR Gerhold KA Bifolck-Fisher A: TRPA1 is required for histamine-independent, Mas-related G protein-coupled receptor-mediated itch. *Nat Neurosci.* 2011;14(5):595–602. 10.1038/nn.2789 21460831 PMC3181150

[12] Wilson SR Nelson AM Batia L: The ion channel TRPA1 is required for chronic itch. *J Neurosci.* 2013;33(22):9283–94. 10.1523/JNEUROSCI.5318-12.2013 23719797 PMC3752436

[13] Gomes LO Hara DB Rae GA: Endothelin-1 induces itch and pain in the mouse cheek model. *Life Sci.* 2012;91(13–14):628–33. 10.1016/j.lfs.2012.03.020 22483687

[14] Kido-Nakahara M Buddenkotte J Kempkes C: Neural peptidase endothelin-converting enzyme 1 regulates endothelin 1-induced pruritus. *J Clin Invest.* 2014;124(6):2683–95. 10.1172/JCI67323 24812665 PMC4038561

[15] Devigili G Eleopra R Pierro T: Paroxysmal itch caused by gain-of-function Nav1.7 mutation. *Pain.* 2014;155(9):1702–7. 10.1016/j.pain.2014.05.006 24820863

[16] Lee JH Park CK Chen G: A monoclonal antibody that targets a Na _V_1.7 channel voltage sensor for pain and itch relief. *Cell.* 2014;157(6):1393–404. 10.1016/j.cell.2014.03.064 24856969 PMC4098795

[17] Storan ER O'Gorman SM McDonald ID: Role of cytokines and chemokines in itch. *Handb Exp Pharmacol.* 2015;226:163–76. 10.1007/978-3-662-44605-8_9 25861779

[18] Singer EM Shin DB Nattkemper LA: IL-31 is produced by the malignant T-cell population in cutaneous T-Cell lymphoma and correlates with CTCL pruritus. *J Invest Dermatol.* 2013;133(12):2783–5. 10.1038/jid.2013.227 23698099

[19] Nattkemper LA Martinez-Escala ME Gelman AB: Cutaneous T-Cell Lymphoma and Pruritus: the Expression of IL-31 and its Receptors in the Skin. *Acta Derm Venereol.* 2016. 10.2340/00015555-2417 27001482

[20] Tey HL Cao T Nattkemper LA: Pathophysiology of pruritus in primary localized cutaneous amyloidosis. *Br J Dermatol.* 2016;174(6):1345–50. 10.1111/bjd.14391 26748444

[21] Arai I Tsuji M Takeda H: A single dose of interleukin-31 (IL-31) causes continuous itch-associated scratching behaviour in mice. *Exp Dermatol.* 2013;22(10):669–71. 10.1111/exd.12222 24079740

[22] Arai I Tsuji M Miyagawa K: Repeated administration of IL-31 upregulates IL-31 receptor A (IL-31RA) in dorsal root ganglia and causes severe itch-associated scratching behaviour in mice. *Exp Dermatol.* 2015;24(1):75–8. 10.1111/exd.12587 25381841

[23] Sun YG Chen ZF: A gastrin-releasing peptide receptor mediates the itch sensation in the spinal cord. *Nature.* 2007;448(7154):700–3. 10.1038/nature06029 17653196

[24] Mishra SK Hoon MA: The cells and circuitry for itch responses in mice. *Science.* 2013;340(6135):968–71. 10.1126/science.1233765 23704570 PMC3670709

[25] Liu XY Wan L Huo FQ: B-type natriuretic peptide is neither itch-specific nor functions upstream of the GRP-GRPR signaling pathway. *Mol Pain.* 2014;10:4. 24438367 10.1186/1744-8069-10-4 PMC3930899

[26] Ross SE Mardinly AR McCord AE: Loss of inhibitory interneurons in the dorsal spinal cord and elevated itch in *Bhlhb5* mutant mice. *Neuron.* 2010;65(6):886–98. 10.1016/j.neuron.2010.02.025 20346763 PMC2856621

[27] Kardon AP Polgár E Hachisuka J: Dynorphin acts as a neuromodulator to inhibit itch in the dorsal horn of the spinal cord. *Neuron.* 2014;82(3):573–86. 10.1016/j.neuron.2014.02.046 24726382 PMC4022838

[28] Bourane S Duan B Koch SC: Gate control of mechanical itch by a subpopulation of spinal cord interneurons. *Science.* 2015;350(6260):550–4. 10.1126/science.aac8653 26516282 PMC4700934

[29] Shiratori-Hayashi M Koga K Tozaki-Saitoh H: STAT3-dependent reactive astrogliosis in the spinal dorsal horn underlies chronic itch. *Nat Med.* 2015;21(8):927–31. 10.1038/nm.3912 26193341

[30] Liu T Han Q Chen G: Toll-like receptor 4 contributes to chronic itch, alloknesis, and spinal astrocyte activation in male mice. *Pain.* 2016;157(4):806–17. 10.1097/j.pain.0000000000000439 26645545 PMC4946956

[31] Zhang Y Dun SL Chen YH: Scratching activates microglia in the mouse spinal cord. *J Neurosci Res.* 2015;93(3):466–74. 10.1002/jnr.23501 25354468 PMC4293236

[32] Zhang Y Yan J Hu R: Microglia are involved in pruritus induced by DNFB via the CX3CR1/p38 MAPK pathway. *Cell Physiol Biochem.* 2015;35(3):1023–33. 10.1159/000373929 25661672

[33] Torigoe K Tominaga M Ko KC: Intrathecal Minocycline Suppresses Itch-Related Behavior and Improves Dermatitis in a Mouse Model of Atopic Dermatitis. *J Invest Dermatol.* 2016;136(4):879–81. 10.1016/j.jid.2015.12.037 26827761

[34] Akiyama T Carstens MI Ikoma A: Mouse model of touch-evoked itch (alloknesis). *J Invest Dermatol.* 2012;132(7):1886–91. 10.1038/jid.2012.52 22418875 PMC3375351

[35] Bin Saif GA Papoiu AD Banari L: The pleasurability of scratching an itch: a psychophysical and topographical assessment. *Br J Dermatol.* 2012;166(5):981–5. 10.1111/j.1365-2133.2012.10826.x 22242789 PMC3335970

[36] Mochizuki H Tanaka S Morita T: The cerebral representation of scratching-induced pleasantness. *J Neurophysiol.* 2014;111(3):488–98. 10.1152/jn.00374.2013 24155004

[37] Papoiu AD Nattkemper LA Sanders KM: Brain's reward circuits mediate itch relief. a functional MRI study of active scratching. *PLoS One.* 2013;8(12):e82389. 10.1371/journal.pone.0082389 24324781 PMC3855767

[38] Schut C Mollanazar NK Kupfer J: Psychological Interventions in the Treatment of Chronic Itch. *Acta Derm Venereol.* 2016;96(2):157–61. 10.2340/00015555-2177 26073701

[39] Beck LA Thaçi D Hamilton JD: Dupilumab treatment in adults with moderate-to-severe atopic dermatitis. *N Engl J Med.* 2014;371(2):130–9. 10.1056/NEJMoa1314768 25006719

[40] Nemoto O Furue M Nakagawa H: The first trial of CIM331, a humanized antihuman interleukin-31 receptor A antibody, in healthy volunteers and patients with atopic dermatitis to evaluate safety, tolerability and pharmacokinetics of a single dose in a randomized, double-blind, placebo-controlled study. *Br J Dermatol.* 2016;174(2):296–304. 10.1111/bjd.14207 26409172

[41] Jarnagin K Chanda S Coronado D: Crisaborole Topical Ointment, 2%: A Nonsteroidal, Topical, Anti-Inflammatory Phosphodiesterase 4 Inhibitor in Clinical Development for the Treatment of Atopic Dermatitis. *J Drugs Dermatol.* 2016;15(4):390–6. 27050693

[42] Farahnik B Beroukhim K Nakamura M: Anti-IL-17 Agents for Psoriasis: A Review of Phase III Data. *J Drugs Dermatol.* 2016;15(3):311–6. 26954316

[43] Sobell JM Foley P Toth D: Effects of Apremilast on Pruritus and Skin Discomfort/Pain Correlate With Improvements in Quality of Life in Patients With Moderate to Severe Plaque Psoriasis. *Acta Derm Venereol.* 2016;96(4):514–20. 10.2340/00015555-2360 26837052

[44] Inui S: Nalfurafine hydrochloride to treat pruritus: a review. *Clin Cosmet Investig Dermatol.* 2015;8:249–55. 10.2147/CCID.S55942 26005355 PMC4433050

[45] Cowan A Kehner GB Inan S: Targeting Itch with Ligands Selective for κ Opioid Receptors. *Handb Exp Pharmacol.* 2015;226:291–314. 10.1007/978-3-662-44605-8_16 25861786

[46] Ständer S Luger TA: NK-1 Antagonists and Itch. *Handb Exp Pharmacol.* 2015;226:237–55. 10.1007/978-3-662-44605-8_14 25861784

